# Carotid reconstruction in patients operated for malignant head and neck neoplasia

**DOI:** 10.1590/S1516-31802002000500003

**Published:** 2002-09-02

**Authors:** Kenji Nishinari, Nelson Wolosker, Guilherme Yazbek, Luiz Caetano Malavolta, Antônio Eduardo Zerati, Luiz Paulo Kowalski

**Keywords:** Head and neck neoplasms, Carotid arteries, Neck neoplasms, Neoplasia, Carotid artery diseases, Carotid, Artery, Vascular, Resection, Arterial, Grafting, Neoplasia, Cabeça Pescoço, Artéria, Carótida, Ressecção, Vascular, Enxerto, Arterial

## Abstract

**CONTEXT:**

Patients with malignant head and neck neoplasia may present simultaneous involvement of large vessels due to the growth of the tumoral mass. The therapeutic options are chemotherapy, radiotherapy, surgery or combined treatments.

**OBJECTIVE:**

To analyze the result of surgical treatment with carotid reconstruction in patients with advanced malignant head and neck neoplasia.

**DESIGN:**

Prospective.

**SETTING:**

Hospital do Câncer A.C. Camargo, São Paulo, Brazil.

**PARTICIPANTS:**

Eleven patients operated because of advanced malignant head and neck neoplasia that was involving the internal and/or common carotid artery.

**MAIN MEASUREMENTS:**

By means of clinical examination, outpatient follow-up and duplex scanning, we analyzed the patency of the carotid grafts, vascular and non-vascular complications, disease recurrence and survival of the patients.

**RESULTS:**

Six patients (54.5%) did not present any type of complication. There was one vascular complication represented by an occlusion of the carotid graft with a cerebrovascular stroke in one hemisphere. Non-vascular complications occurred in five patients (45.5%). During the follow-up, eight patients died (72.7%), of whom seven had loco-regional tumor recurrence and one had pulmonary and hepatic metastases (at an average of 9 months after the operation). Seven of these patients presented functioning grafts. The three patients still alive have no tumor recurrence and their grafts are functioning (an average of 9 months has passed since the operation).

**CONCLUSIONS:**

Patients with advanced malignant head and neck neoplasia involving the carotid artery that are treated surgically present a prognosis with reservations. When the internal and/or common carotid artery is resected en-bloc with the tumor, arterial reconstruction must be performed. The long saphenous vein is a suitable vascular substitute.

## RESUMO

**CONTEXTO:**

Os pacientes portadores de neoplasia maligna de cabeça e pescoço podem apresentar acometimento simultâneo de grandes vasos devido ao crescimento da massa tumoral. As opções terapêuticas são a quimioterapia, radioterapia, cirurgia ou tratamento combinado. Quando o tratamento cirúrgico é indicado e a veia jugular interna é acometida, normalmente é ressecada sem reconstrução, pois geralmente não há repercussões clínicas importantes. Porém, quando a artéria carótida interna e/ou comum são acometidas, a ressecção sem revascularização normalmente leva a índices de complicações neurológicas elevadas, motivo pelo qual deve ser realizado enxerto arterial.

**OBJETIVO:**

Analisar o resultado do tratamento cirúrgico com reconstrução carotídea dos pacientes portadores de neoplasia maligna avançada de cabeça e pescoço.

**TIPO DE ESTUDO:**

Prospectivo.

**LOCAL:**

Hospital do Câncer A.C. Camargo, São Paulo, SP, Brasil.

**PARTICIPANTES:**

11 pacientes operados por neoplasia maligna avançada de cabeça e pescoço acometendo artéria carótida interna e/ou comum.

**PRINCIPAIS VARIÁVEIS:**

Por meio de exame clínico, seguimento ambulatorial e mapeamento dúplex, analisamos a perviedade dos enxertos carotídeos, as complicações vasculares e não-vasculares, recorrência da doença e sobrevida dos pacientes.

**RESULTADOS:**

Seis pacientes (54,5%) não apresentaram nenhum tipo de complicação. Houve uma complicação vascular representada por oclusão do enxerto carotídeo com acidente vascular cerebral hemisférico. As complicações não-vasculares ocorreram em cinco pacientes (45,5%). Durante o seguimento, oito pacientes faleceram (72,7%), sendo sete com recidiva tumoral loco-regional e um com metástases pulmonares e hepáticas (média de nove meses após a operação). Sete desses pacientes apresentavam enxerto funcionante. Os três pacientes vivos encontram-se sem recidiva tumoral e com enxertos funcionantes (média de nove meses).

**CONCLUSÕES:**

Os pacientes com neoplasia maligna avançada de cabeça e pescoço acometendo artéria carótida tratados cirurgicamente apresentam prognóstico reservado. Quando a artéria carótida interna e/ou comum é ressecada em bloco com o tumor, a reconstrução arterial deve ser realizada, sendo a veia safena magna um substituto vascular adequado.

## INTRODUCTION

Patients with malignant head and neck neoplasia may present large vessels that have been involved through the invasion of the vessel wall by the adjacent tumor or vessels that have been confined due to the growth of the tumoral mass. The therapeutic options for these cases of advanced neoplasia are: chemotherapy, radiotherapy, surgery or combined treatments. Chemotherapy and radiotherapy used in isolation or in combination rarely provide a cure. Cervical control that associates surgery with radiotherapy (teleradiotherapy or brachy-therapy) is the therapeutic alternative that gives the best possibility for locoregional control of the disease over the long term.^1^

When surgical treatment is indicated and the internal jugular vein has been involved, resection is normally performed without reconstruction. There are no significant repercussions from this, especially if the contralateral vein is preserved.^2,3^ However, when the common or internal carotid arteries have been involved, resection without revascularization usually leads to high rates of neurological complications.^4,5,6^

The objective of this study was to analyze the result of the surgical treatment of malignant head and neck neoplasia with the associated carotid reconstruction, taking into consideration the patency of the reconstruction, vascular and non-vascular complications, recurrence and survival.

## METHODS

Eleven patients operated because of advanced malignant head and neck neoplasia that had involved the internal and/or common carotid artery were followed up prospectively over the period from January 1997 to January 2001. In one patient, in addition to the common carotid artery, there had been simultaneous involvement of the subclavian artery and vein. Among the patients with advanced neoplasia, surgical treatment was only indicated for those whose clinical examination demonstrated the presence of a mobile tumor mass and whose computerized tomography did not demonstrate signs of invasion of the base of the skull. All the patients were males, with ages ranging from 38 to 75 years (an average of 58 years). All the patients were smokers and two were alcoholics. Four of them presented systemic arterial hypertension. With regard to prior treatment for the neoplasia, only three patients had not undergone any type of therapy. Eight patients had already undergone operation, eight had had radiotherapy treatment and five chemo-therapy. The anatomical sites of the tumor were: larynx (4), tongue (3), pharynx (1), vallecula (1), supra-clavicular region/base of the neck (1). In one patient, the primary site of the neoplasia was not identified (cervical metastasis with concealed primary tumor) (Table 1).

**Table 1:** Demographic characteristics, tumor site and histology, previous treatment, vascular reconstruction and complications of eleven male patients operated for head and neck cancer

Patient Initials	Age	Site	Type	Previous treatment	Substitute	DCC (minutes)	Vascular complication
CCS	69	Pharynx	SCC	-	Saphenous	26	Occlusion
OT	61	Larynx	SCC	SUR + RT + CT	Saphenous	22	-
ER	54	Larynx	SCC	SUR + RT + CT	Saphenous	23	-
LHBL	54	Tongue	SCC	SUR + RT + CT	Saphenous	29	-
JRN	57	Vallecula	SCC	-	Saphenous	26	-
LAN	60	Larynx	SCC	-	Saphenous	22	-
MJR	38	Tongue	SCC	SUR + RT	Saphenous	28	-
				+ CT			
ALB	46	Concealed	SCC	SUR + RT	Saphenous	28	-
				+ CT			
CAS	65	Neck skin	CBC	SUR + RT	PTFE	18	-
AHRC	65	Larynx	SCC	SUR + RT	Saphenous	25	-
MP	75	Tongue	SCC	SUR + RT	Saphenous	27	-

*SCC = squamous cell carcinoma; CBC = basal cell carcinoma; SUR = surgery; RT = radiotherapy; CT = chemotherapy; PTFE = expanded polytetrafluoroethylene prosthesis; DCC = duration of carotid clamping.*

Upon clinical vascular examination, only the patient with the compromised subclavian vessels and carotid artery presented an alteration. This consisted of an absence of arterial pulse in the upper left limb, associated with lymphedema.

With regard to complementary examinations, all the patients had computerized tomography, in which the tumoral mass was always shown to be lacking a cleavage plane or agglomerating the resected vessels. Four patients presented arteriography of the carotid system that demonstrated adequate collateral circulation and absence of significant bilateral carotid stenosis. In the other seven patients, the duplex scanning did not demonstrate significant alterations in the bilateral carotid flow. In the patient with absence of arterial pulse in the upper left limb, angiography demonstrated obstruction of the subclavian artery and refilling of the axillary artery.

*En-bloc* resection of the vessels affected by the tumor was indicated by the head and neck surgeon on the basis of the operative findings, which brought out evidence of agglomeration or adherence to the tumoral mass. The procedure was indicated in the cases where vascular resection would be appropriate from an oncological point of view and there were no other areas of tumor that were not resectable or where the resectability would be in doubt.

After the definition of the carotid resection, the vascular surgery team proceeded with the removal of the long saphenous vein in 10 cases. In one case, the staff decided to utilize an expanded polytetrafluoroethylene (PTFE) prosthesis with an external support. To perform the vascular grafting, all the patients were given heparin anticoagulant via the endovenous route.

The clamping and sectioning of the vessels was performed as the final step of the *en-bloc* resection, after freeing all the tumor material and removing the long saphenous vein, with the aim of minimizing the duration of cerebral ischemia. Only in the patient with both the carotid artery and the subclavian vessels involved could this sequence not be followed. This led us to utilize a temporary Pruitt-Inahara shunt during the resection of the tumor. The duration of carotid clamping varied from 18 to 29 minutes, with an average of 25 minutes.

In ten patients a common carotid to internal carotid graft was performed with the autologous saphenous not inverted. In the patient with simultaneous involvement of the carotid and subclavian vessels, three grafts were performed using a PTFE prosthesis: a common carotid to common carotid graft with a lateral lead to the axillary artery, and an axillary vein to innominate vein graft.

All the patients required a myocutaneous flap, to cover the vascular graft and the bloodstained area.

The histological types encountered were ten squamous cell carcinomas and one basal cell carcinoma. The anatomopathological study demonstrated invasion of the vessel wall in seven cases (63.6 %).

The follow-up of the patients was done by means of four-monthly outpatient returns, at which clinical examination and duplex scanning were performed in order to study the patency of the grafts.

## RESULTS

Six patients (54.5%) did not present any type of complication during the follow-up. Ten patients did not present a vascular complication. Only one patient presented occlusion of the carotid graft, which occurred in the immediate postoperative period, leading to severe clinical repercussions: a cerebrovascular stroke in one hemisphere with sequelae of hemiparesis and aphasia. This patient also presented partial necrosis of the flap and a pharyngeal-cutaneous fistula. There were no complications at the site from which the long saphenous vein was removed.

Non-vascular complications occurred in five patients (45.5%), of whom one patient presented two complications simultaneously. The complications were: cervical cellulitis (2), partial necrosis of the flap (2), pharyngeal-cutaneous fistula (1) and partial dehiscence of the flap (1). The cellulitis was successfully treated using systemic antibiotic therapy. The necrosis of the flaps required debridement of the devitalized tissue, and these cases evolved satisfactorily. The partial dehiscence required the flap to be resutured. Only the pharyngeal-cutaneous fistula was not resolved using conservative or surgical treatment.

During the follow-up, eight patients died (72.7%), of whom seven had locoregional tumor recurrence and one had pulmonary and hepatic metastases. Seven of these patients presented functioning grafts. The length of time between the operation and death ranged from 4 to 20 months, with an average of 9 months.

The patients that are still alive are 5, 10 and 13 months (an average of 9 months) into the postoperative period, without tumor recurrence and with functioning grafts.

## DISCUSSION

The surgical treatment of patients with malignant head and neck neoplasia that has involved the carotid artery is debatable, considering the reservations that exist for the long-term prognosis.^7,8^ The main objective is the locoregional control of the disease^9,10,11^ and a possible improvement in the quality of life.

In these cases, when performing the re-section of the tumor, the carotid artery can be approached in three ways: sub-adventitial dissection,^12,13^ carotid resection with ligature of the stumps^4,5,6^ or carotid resection with reconstruction of the flow.^7,11,14^

Resection of the tumor with sub-adventitial dissection is possible in some cases, when there is no firm adherence of the tumor to the carotid. With this technique, the cerebral blood flow is not interrupted, although the presence of microscopic tumor invasion of the vessel wall causes a high local recurrence rate for the disease.^13,15^ In addition there is a risk of arterial rupture because of erosion of the wall after a proliferation of tumor cells, which may be increased after adjuvant radiotherapy.^16^ In cases of superficial invasion with the possibility of shaving the tumor from the artery, the rotation of a thin muscle flap (scapula elevator) for vessel protection and implantation of catheters for brachytherapy is a viable alternative.

When carotid resection is performed *en-bloc* with the tumor so as to obtain an adequate oncological margin, two alternatives are possible: ligature of the vessel stumps or arterial reconstruction. Arterial ligature without reconstruction has already been attempted, although the results are considered unacceptable. This technique has high neurological complication rates^9^ (17% to 45%), even when preoperative tests (balloon occlusion test,^17^ arteriography^4^ and oculoplethysmography)^18^ and intraoperative tests (measurement of reflux pressure)^19^ demonstrate the presence of adapted collateral circulation. The reconstruction of carotid flow after its resection is the best procedure, even after considering the rates of ischemic neurological complications, which can range from 7% to 20%.^8,20^

In this study, among the patients with advanced neoplasia whose carotid arteries were probably compromised, surgical treatment was only indicated for those whose clinical examination demonstrated the presence of a mobile tumor mass and whose computerized tomography did not demonstrate signs of invasion of the base of the skull. The mobility of the mass and the images obtained from the tomography were considered indicative of the presence of a distal cervical carotid stump that was free of disease, thus making it possible to perform the grafting, which was confirmed in all the cases. We did not perform any carotid ligature.

The reconstruction of the carotid flow is normally performed via the interposition of a bridge between the vessel stumps by utilizing an autologous^7,10,21,22^ or synthetic substitute.^8,23^ Because of the increased risk of local infection due to the exposure of mucosa of the airway/digestive tracts, the presence of tracheostomy and irradiated tissue, autologous substitutes are preferred, since they have greater biological compatibility. The long saphenous vein and the superficial femoral artery are the ones most utilized. The advantage of the superficial femoral artery in relation to the long saphenous vein is the greater resistance of its walls.^21^ However, when this substitute is utilized, it is necessary to remove it from its substrate and perform the artery bridge using a prosthesis, with additional risks for the patient.^20^ The advantages of the long saphenous vein^7,10^ is that it is easy to obtain and its caliber is suitable. For these reasons, it was utilized for 10 of our patients. In the single case where we chose to use a PTFE prosthesis, there was no exposure of mucosa or tracheostomy. In addition, this substitute favored the construction of an endto-side anastomosis (carotid to axillary) out of the substrate, thereby avoiding a second clamping of the carotid, which would have caused a longer period of cerebral ischemia.

The intraoperative monitoring and cerebral protection methods that are most used are: carotid stump pressure,^19^ the appearance of the reflux, electroencephalography,^24^ transcranial Doppler,^25^ locoregional anesthesia,^26^ temporary shunt,^27^ utilization of high doses of barbiturates,^28^ induced arterial hyper-tension and hypercapnia.^29^ There is much controversy in the literature regarding these methods as protective factors in carotid surgery, as none of them is totally reliable and their routine use does not impede the occurrence of neurological complications.^4,7,30^

All the patients were operated under general anesthesia with appropriate control of arterial pressure. The carotid reflux was bright red and squirted out in all cases. There were three reasons why we did not utilize temporary shunts on a routine basis: no patient presented significant contralateral obstructive carotid disease; the reflux observed was satisfactory in all cases; and the clamping and sectioning of the carotid were performed as the final step before vascular reconstruction, resulting in an average duration of ischemia that was less than 30 minutes. Only in the case in which both the carotid and subclavian vessels were involved was the carotid sectioned at an early stage. In this case, the vessels were the cause of technical difficulties in the resection of the tumoral mass, and so the carotid was sectioned before the mass was totally extricated. For this reason, we decided to utilize a temporary shunt that lasted 50 minutes.

On the basis of studies of carotid endarterectomy and carotid revascularization associated with resection of neoplasia, it is thought that neurological complications related to carotid revascularization are caused by prolonged transitory hypoperfusion^31^ (duration of carotid clamping greater than 30 minutes) and by thromboembolic phenomena.^19,32^ The only vascular complication in our sample occurred in the immediate postoperative period, after thrombosis of the carotid graft, leading to hemispheric ischemia with severe symptomatology and sequelae.

Patients with advanced malignant head and neck neoplasia and a compromised carotid artery who are treated surgically present prognoses with reservations.^33^ This was observed in our sample, given that eight of the eleven patients died within one year of the operation. The morbidity and mortality rates are considerable and the vascular intervention needs to be done at the same time so that the neurological complications are minimized, without interfering in the patient's survival.

## CONCLUSIONS

Patients with advanced malignant head and neck neoplasia that has involved the carotid arteries who are treated surgically present prognoses with reservations^1^ When the internal and/or common carotid artery is resected *en-bloc* with the tumor, arterial reconstruction must be performed, with the long saphenous vein forming a suitable vascular substitute.
